# Omics data integration in computational biology viewed through the prism of machine learning paradigms

**DOI:** 10.3389/fbinf.2023.1191961

**Published:** 2023-08-04

**Authors:** Aziz Fouché, Andrei Zinovyev

**Affiliations:** ^1^ Institut Curie, PSL Research University, Paris, France; ^2^ Institut National de la Santé et de la Recherche Médicale, Paris, France; ^3^ CBIO-Centre for Computational Biology, ParisTech, PSL Research University, Paris, France; ^4^ Ecole Normale Supérieure Paris-Saclay, Cachan, France; ^5^ In Silico R&D, Evotec, Toulouse, France

**Keywords:** single-cell, data integration, machine learning, batch effect, multi-omics

## Abstract

Important quantities of biological data can today be acquired to characterize cell types and states, from various sources and using a wide diversity of methods, providing scientists with more and more information to answer challenging biological questions. Unfortunately, working with this amount of data comes at the price of ever-increasing data complexity. This is caused by the multiplication of data types and batch effects, which hinders the joint usage of all available data within common analyses. Data integration describes a set of tasks geared towards embedding several datasets of different origins or modalities into a joint representation that can then be used to carry out downstream analyses. In the last decade, dozens of methods have been proposed to tackle the different facets of the data integration problem, relying on various paradigms. This review introduces the most common data types encountered in computational biology and provides systematic definitions of the data integration problems. We then present how machine learning innovations were leveraged to build effective data integration algorithms, that are widely used today by computational biologists. We discuss the current state of data integration and important pitfalls to consider when working with data integration tools. We eventually detail a set of challenges the field will have to overcome in the coming years.

## 1 Introduction

This last decade has witnessed a sharp increase in the amount and complexity of data produced for cellular biology, thanks to an ever-growing number of bulk and single-cell profiling assays. These technologies allowed scientists to study heterogeneous cell populations through many biological feature spaces (or *modalities*) such as mRNA expression (Klein et al., 2015; Macosko et al., 2015), DNA methylation (Guo et al., 2013) and chromatin accessibility (Buenrostro et al., 2015a; Buenrostro et al., 2015b), and protein abundance (Aebersold and Mann, 2003; Westermeier and Marouga, 2005; Tibes et al., 2006). These assays can be carried out either in bulk, which yields for each sample a single averaged molecular profile, or at the single-cell level, which provides an exquisite insight into cell states and types present in the cell population. In particular, carrying out biological assays at the single-cell level snapshots cells at various points of a dynamical process, which can then be leveraged for various applications such as lineage tracing (Schiebinger et al., 2019), transcriptional dynamics (La Manno et al., 2018), inference of transcriptional trajectories (Chen H. et al., 2019) and many more.

In addition, during the last few years, there have been several joint assays proposed to profile single cells through several modalities simultaneously, such as scM&T-seq for transcriptome and methylome (Angermueller et al., 2016), sc-GEM for genotype, transcriptome and methylome (Cheow et al., 2016), CITE-seq for transcriptome and surface proteins (Stoeckius et al., 2017), or SNARE-seq for transcriptome and chromatin accessibility (Chen S. et al., 2019). It is also worth mentioning spatial transcriptomics, which yields measurements from a small number of cells in each well while also providing positional information of cells within the biological tissue (Ståhl et al., 2016). Finally, important phenotypical information can be obtained from microscopic imaging data, such as whole slide imaging (Pantanowitz et al., 2011).

Hand-to-hand with the surge of biological modalities, there has been an explosion in the number of available datasets helped by various scientific initiatives to make biological data more easily available (Conesa and Beck, 2019); among these initiatives, one can mention atlases of entire organisms such as the Tabula Muris (Schaum et al., 2018) and Human (Tabula Sapiens Consortium et al., 2022) Consortia. We would also like to talk about disease-based atlas such as The Cancer Genome Atlas (TGCA) database (Weinstein et al., 2013), and the IMMUcan database (Camps et al., 2023) which provides an exquisite insight into the nature of tumor microenvironment. When tackling difficult biological questions, using data gathered across different sources or modalities is enticing. On the one hand, combining data from different sources helps to provide a comprehensive view of the biological object of interest. For example, it can facilitate the discovery of rare but relevant cell types or states, or help quantify the relative abundance of cell types across a collection of biological samples. On the other hand, having different modalities at their disposal allows scientists to link them together, possibly leading to exciting mechanistic discoveries. Finally, there can be an emergent property where analyzing a biological object through several modalities simultaneously could yield superior information compared to analyzing each modality individually.

Unfortunately, there are several obstacles to overcome before data from several sources and modalities can be used within an analysis pipeline. First, the multiplicity of sources comes at the price of all sorts of batch effects, as datasets can come from different replicas, technologies, individuals, or even species. Then, combining datasets containing measurements from different modalities is a major computational challenge, especially when samples are not linked across datasets, as there is no trivial common space to embed samples together. Therefore, there is a real need for methods and tools that would be able to tie together biological datasets across datasets (or *batches*) and modalities. In this review, we investigate this question through the prism of machine learning paradigms, and present how a few of these concepts are today widely used within popular, state-of-the-art data integration methods.

## 2 Data integration links biological datasets across batches or modalities

**FIGURE 1:**
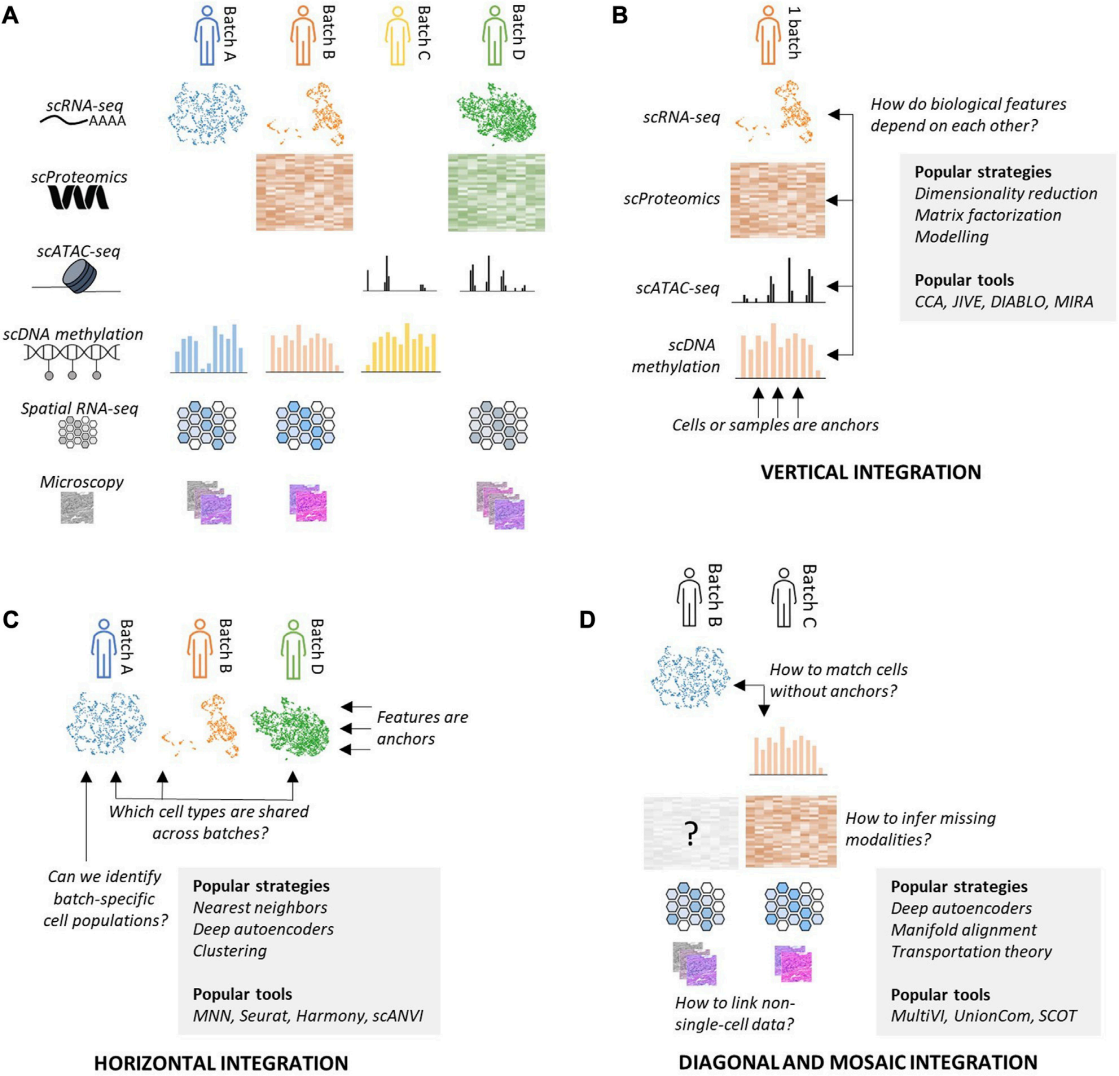
Data integration describes a set of problems aiming to tie together data across different origins or modalities. **(A)** A biological object can be profiled through multiple batches (columns) and modalities (rows), and not all batches necessarily contain measurements for all modalities. **(B)** Vertical integration (VI) consists in using cells or samples as anchors to deduce links between features across modalities. **(C)** Horizontal integration (HI) consists in using overlapping features as anchors to jointly analyze data coming from different sources. **(D)** Diagonal integration (DI) consists in embedding together several batches with non-overlapping modalities. Mosaic integration (MI) is the problem of missing modalities inference.

Data integration describes a set of problems that represent different facets of the question of tying together biological datasets across batches and modalities: *vertical*, *horizontal*, *diagonal* and *mosaic* integration (Argelaguet et al., 2021), which indicate the nature of anchors that exist between datasets (Figure 1A).

In vertical integration (VI), each dataset contains a set of measurements carried out on the same set of samples (separate bulk experiments with matched samples in different modalities or single-cells measured through joint assays) (Figure 1B). VI identifies links between biological features, such as scRNA-seq transcript counts and scATAC-seq peaks, which can help formulate mechanistic hypotheses across modalities. VI methods usually rely on dimensionality reduction, matrix factorization, or modeling. Some can be endowed with additional biological knowledge, such as pathway data and functional interaction between features across modalities.

Horizontal integration (HI) describes the complementary task where several datasets have been acquired in the same biological modality, allowing multiple batches to be expressed within a common features space (Figure 1C). HI’s primary use is to correct batch effects between datasets that can be explained by experimenter variation, different sequencing technologies, or inter-individual biological specificities (e.g., species, sex, or ethnicity). HI has been a very popular research topic for the last few years, and many HI tools have been proposed to this day. They can rely on a large variety of computational paradigms such as nearest neighbors, clustering, deep neural networks, matrix factorization, manifold alignment, and many more. Some tools may require additional priors, such as selecting a reference dataset or having access to cell types as labels.

When no trivial anchoring exists between datasets, diagonal (DI) or mosaic integration (MI) formalisms must be used. DI describes the framework where each dataset is measured in a different biological modality, while MI allows pairs of datasets to be measured in overlapping modalities (Figure 1D). DI and MI are the most challenging facets of data integration and are subject to active research. Methods proposed to perform DI and MI usually rely on advanced machine learning paradigms capable of high levels of abstraction, such as deep neural networks, manifold alignment, or transport theory. Some tools operate in a completely unsupervised fashion, while others require additional information to help them bridge the gap between modalities.

Data integration of biological data is tightly related to several machine learning topics such as domain adaptation (Pan et al., 2010; You et al., 2019; Farahani et al., 2021), data fusion (Castanedo, 2013; Gao et al., 2020) and manifold alignment (Wang et al., 2011). Therefore, it is unsurprising to observe strategies leveraging similar machine learning paradigms such as supervised dimensionality reduction, matrix factorization, nearest neighbors, optimal transport, or deep autoencoders. Interestingly, new methods in all these domains go hand-to-hand with advances in machine learning, with many recent methods featuring advanced machine learning concepts. This is arguably a natural evolution as data complexity and quantity increase, which motivates the need for more powerful models capable of increased levels of abstraction.

## 3 Horizontal integration (HI) links batches anchored by their common modality

Horizontal integration (HI) describes the situation where several batches are all gathered in a common modality with overlapping feature spaces. It is worth noting that depending on the tool, there may only suffice that each pair of datasets contains an overlapping feature space (e.g., dataset A containing features $\left\{f_{1},f_{2}\right\}$ , dataset B containing features $\left\{f_{1},f_{3}\right\}$ and dataset C containing features $\left\{f_{2},f_{3}\right\}$ ). HI is a convenient framework in which cells can directly be compared across different batches due to their feature space overlap, which allows the use of natural concepts such as distances, neighborhoods, or similarity measures. Many tools have been proposed to tackle HI, and we gathered a non-exhaustive list of them in (Table 1). As we can see, these methods use various strategies to identify similar cells across batches and embed cells into a joint space. Some require additional information, such as reference datasets or cell labels. The remainder of this section is devoted to describing the main computational principles and machine learning paradigms HI methods rely on and providing some rationale and guidelines about each of them.

**TABLE 1:** A non-exhaustive list of horizontal integration (HI) tools aiming to jointly embed single-cell datasets measured in the same modality into a common space. BA, Bayesian; NN, Nearest Neighbors; DAE, Deep Autoencoders; DR, Dimensionality Reduction; IC, Iterative Clustering; MF, Matrix Factorization; MA, Manifold Alignment; RE, Regression; FR, Framework.

Tool	Strategy	Input	Output	Year	References
ComBAT	BA	RNA-seq	Gene space	2007	Johnson et al. (2007)
MNN	NN	RNA-seq	Gene space	2018	Haghverdi et al. (2018)
scmap	NN	RNA-seq	Clustering	2018	Kiselev et al. (2018)
scvi	DAE	RNA-seq, spatial	Embedding	2018	Lopez et al. (2018)
ingest	DR	RNA-seq	Embedding	2018	Wolf et al. (2018)
CONOS	NN	RNA-seq	Graph	2019	Barkas et al. (2019)
Scanorama	NN	RNA-seq	Embedding	2019	Hie et al. (2019)
scAlign	DAE	RNA-seq	Embedding	2019	Johansen and Quon (2019)
Harmony	CL	RNA-seq	Embedding	2019	Korsunsky et al. (2019)
Seurat v3	NN	RNA-seq	Gene space	2019	Stuart et al. (2019)
LIGER	MF	RNA-seq	Embedding	2019	Welch et al. (2019)
DESC	DAE	RNA-seq	Embedding	2020	Li et al. (2020)
BBKNN	NN	RNA-seq	Graph	2020	Polański et al. (2020)
SpaGE	NN	RNA-seq, spatial	Embedding	2020	Abdelaal et al. (2020)
Tangram	DAE	RNA-seq, spatial	Embedding	2021	Biancalani et al. (2021)
Canek	NN	RNA-seq	Embedding	2022	Loza et al. (2022)
CAPITAL	MA	RNA-seq	Embedding	2022	Sugihara et al. (2022)
SCISSOR	RE	RNA-seq	Graph	2022	Sun et al. (2022)
Transmorph	FR	RNA-seq	Embedding	2022	Fouché et al. (2022)
DAPCA	MF	Any	Embedding	2023	Mirkes et al. (2023)

**FIGURE 2:**
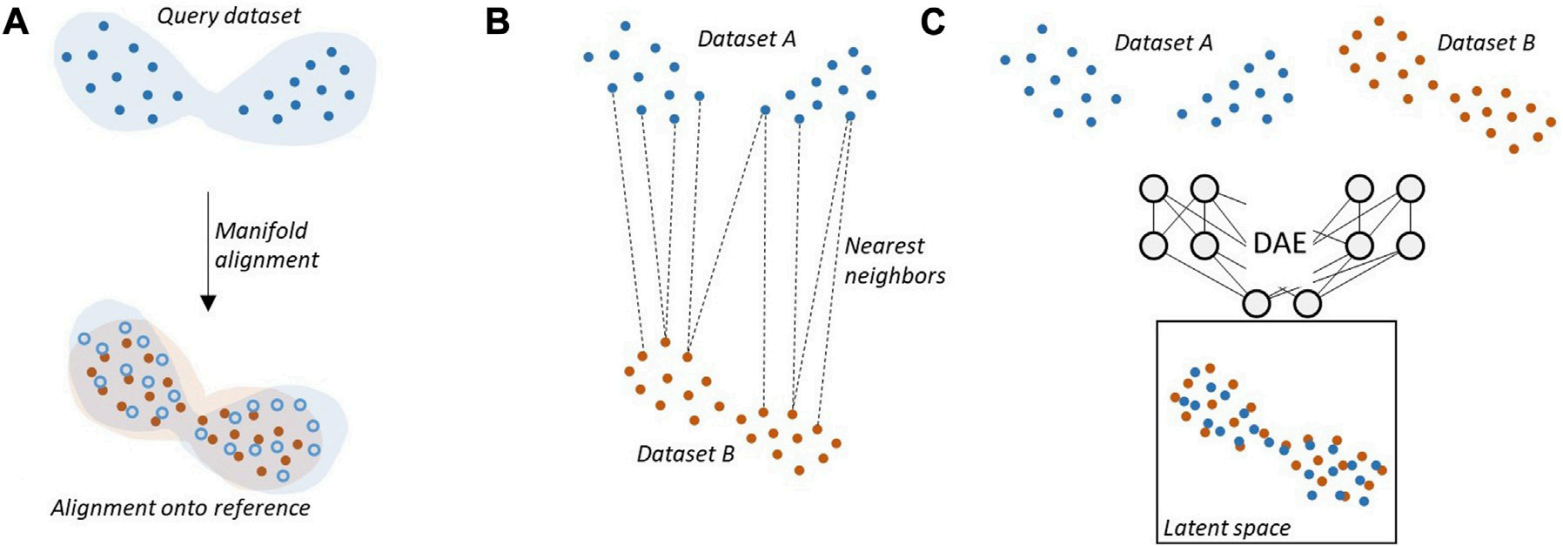
Horizontal integration describes the problem of embedding together datasets measured along the same biological modality. Different types of popular machine learning approaches are commonly used to match similar cells across batches. **(A)** Manifold alignment techniques find the projection that create the optimal overlap between two point clouds. **(B)** Nearest neighbors techniques identifies similar cells across datasets based on a similarity measure. **(C)** Deep autoencoders (AEC) learn a joint latent representation of the data in which batch effects are regressed out.

Many HI methods rely on manifold alignment strategies to integrate batches together (Figure 2A), allowing them to consider the whole data structure instead of matching individual cells. Perhaps the oldest and most natural manifold alignment technique is Procrustes analysis (Gower, 1975), named after the mythical greek thug who cut or stretched his victims so that they fit the length of their bed. This is an old and intuitive machine learning paradigm mostly used for shape alignment that aims at projecting query datasets onto a reference one while only allowing simple transformations (rotation, rescaling, and shifting). Procrustes-based methods are not often used to integrate single-cell data, although some attempts can be found in the literature (Eto et al., 2018). First introduced to infer cell differentiation trajectories (Schiebinger et al., 2019), discrete optimal transport (OT) theory and its extensions (Gromov-Wasserstein, partial OT, unbalanced OT) is the most popular paradigm used for manifold alignment-based HI. It aims to align cells as discrete probability distributions represented as weighted point clouds in a metric space based on pairwise cell-cell cost matrices between batches that are often distance matrices. OT and its extensions have been successfully applied to horizontal and diagonal data integration (Cao et al., 2022b; Demetci et al., 2022). Manifold alignment-based HI is a powerful paradigm, but it can sometimes struggle to solve complex alignment tasks (for instance, when the structure of a dataset presents ambiguous symmetries or when some batches contain specific cell types that must not be aligned).

Another class of HI methods seeks similar cells across batches, operating at the single-cell level rather than at a global level (Figure 2B). Some are based on the nearest neighbors approach like mutual nearest neighbors (MNN) (Haghverdi et al., 2018), CONOS (Barkas et al., 2019), Scanorama (Hie et al., 2019), Seurat (Satija et al., 2015; Butler et al., 2018; Stuart et al., 2019; Hao et al., 2021) that include different integration schemes such as CCA and robust PCA (RPCA), or BBKNN (Polański et al., 2020). All nearest neighbors-based methods rely on the hypothesis that batch effects are almost orthogonal to biological effects, which would allow identifying similar cells across batches through simple orthogonal projection. They then apply various strategies to end up with a joint representation of cells like correction vectors or joint graph construction. These methods tend to work best when facing slight to moderate batch effects and generally fail when batch effects are far from being orthogonal to relevant biological signals. They tend to scale well to large datasets thanks to various optimizations during nearest neighbors computation like nearest neighbors descent (Dong et al., 2011). Another metric-based approach is described in Harmony (Korsunsky et al., 2019), which is probably the most used tool in practice for HI of single-cell data. It uses an iterative algorithm of successive biased clustering across batches and correction. First, cells are clustered across datasets with such a bias that penalizes clusters of cells with a homogeneous batch of origin. Then, cells of a given cluster are pooled towards each other. An optimality criterion is tested at each iteration to assess whether batch mixing is sufficient, using a local purity metric called Local Inverse Simpson’s Index (LISI). Due to its simplicity and availability with both Python and R packages, Harmony is widely used today and still achieves respectable results in benchmarks (Anaissi et al., 2022) despite being limited when facing strong batch effects (Luecken et al., 2022).

Deep autoencoders (DAEs) (and more recently variational autoencoders) have been popular tools in single-cell for a few years already and excel at performing a variety of complex preprocessing tasks, such as dimensionality reduction (Wang and Gu, 2018), or denoising and correcting dropouts (Eraslan et al., 2019), as well as acting as generative models (Trong et al., 2020). DAEs are neural networks that leverage a bottleneck structure to learn a compressed data representation in a low dimensional space, which can then be exploited for various tasks (Figure 2C). DAE is a powerful framework to carry out horizontal data integration with tools such as scvi (Lopez et al., 2018), scAlign (Johansen and Quon, 2019) or DESC (Li et al., 2020). In particular, scANVI, part of the scvi framework, is the top performer tool in the (Luecken et al., 2022) atlas-scale benchmark. DAEs generally have high computational capabilities thanks to the fact to be able to exploit GPU acceleration during training. The main downside of DAEs is the large amounts of data necessary for their training and their lack of interpretability, though there are efforts to improve on the latter point (Svensson et al., 2020; Treppner et al., 2022).

In an attempt to organize these methods into a common framework, we introduced Transmorph (Fouché et al., 2022), an open-source computational framework that allows the user to assemble custom HI pipelines from basic algorithmic blocks. This framework focuses on methods that combine a matching step, identifying similar cells across batches, and an embedding step, where these correspondences are used to generate a joint representation of all datasets. Transmorph also gives access to pre-build HI pipelines, HI quality assessment routines, benchmarking datasets and easy access to other state-of-the-art HI tools such as Harmony (Korsunsky et al., 2019) and scvi (Lopez et al., 2018). We hope to see more initiatives deployed in the next years in this sense to provide frameworks that can help organize the field of HI methods.

Despite the myriad approaches proposed to tackle HI, it remains challenging today to correct strong batch effects. For instance, (Tran et al., 2020; Luecken et al., 2022), showed that if several methods can satisfyingly remove moderate batch effects, integrating datasets across species remains difficult for unsupervised methods which do not require cell labeling information. Also, many methods rely on finding first an overlapping feature space between all datasets, which can be an obstacle when building large atlases combining many batches of varying quality, where the number of common features can shrink drastically. Finally, the problem of selecting appropriate metrics to assess data integration quality is still difficult. Most benchmarks use a mixture of metrics to measure different aspects of the data integration task such as batch mixture, label clustering or topology preservation, depending on the information available:

• Batch mixture metrics such as batch-LISI are commonly used to measure how much the data integration procedure brought cells from different datasets close to one another. These metrics are popular because they do not require additional information, such as cell types or states, and can be used as unsupervised tools. Unfortunately, a good integration does not necessarily imply good batch mixture metrics, as two datasets without overlapping cell types should not be mixed after integration; similarly, projecting all datasets together onto a single point would result in perfect batch mixing, but all the biological information would be lost. For these reasons, even though batch mixture metrics are quite informative and widely used, most benchmarks also include other integration metrics to compensate for these limitations.
• Label clustering metrics, such as normalized mutual information or adjusted Rand index, provide an additional axis to measure data integration quality by assessing if cells of similar type cluster together after integration. Label clustering metrics are usually quite good for controlling the data integration quality if cell types can be identified confidently. The main downside of these metrics is the necessity to have high-confidence cell labels available before integration, which is often not the case (especially as one of the purposes of data integration is to be carried out before clustering and cell type inference).
• Finally, topology preservation metrics assess how data integration has preserved relations between the different cells and penalize cases where cells that were close before integration have been brought far apart by the algorithm (meaning cells that were initially similar but are dissimilar after integration). Topology can be biology-driven by observing the conservation of signals related to specific cell processes, such as cell cycle or other transcriptomic trajectories, or data-driven with algorithms as simple as comparing the *k*-nearest neighbors of a cell before and after integration and penalizing the differences.

Evaluating the quality of a HI can be daunting, as shown by the large variety of metrics that have been developed for it. In practice, we often use a batch mixture metric such as LISI, complemented by a secondary metric that can be either a label clustering metric if high-confidence labels are available and a topology preservation metric otherwise.

## 4 Vertical integration (VI) connects modalities measured in the same cells

Vertical integration (VI) uses several datasets containing individual measurements from the same cells obtained from joint single-cell assays measured through different biological features (e.g., gene expression and chromatin accessibility) to infer relations between the different modalities (Table 2). VI is usually declined into two variants, namely, *local* VI and *global* VI. Local VI identifies links between individual features (such as genes and methylated promoters), and can be used to formulate hypotheses of direct or indirect biological interactions between the omics layers (e.g., gene expression and accessibility of a chromatin region), with methods like LMM (Van Der Wijst et al., 2018) or Spearman’s rank correlation coefficient (Cuomo et al., 2020). On the other hand, global VI links features across different modalities via global factors that can be related to biological processes (e.g., identifying a group of genes and chromatin regions to correspond to proliferation activity).

**TABLE 2:** A non-exhaustive list of global vertical integration (VI) tools that can be used to learn relations between features across modalities from joint single-cell assays. FC, Feature Correlation; MD, Matrix Decomposition; NN, Nearest Neighbors; DAE, Deep Autoencoders; TM, Topic Modelling.

Tool	Strategy	Input	Year	References
CCA	FC	Any	1936	Hotelling (1992)
RGCCA	FC	Any	2011	Tenenhaus and Tenenhaus (2011)
JIVE	MD	Any	2013	Lock et al. (2013)
SGCCA	FC	Any	2014	Tenenhaus et al. (2014)
MOFA	MD	Any	2018	Argelaguet et al. (2018); Argelaguet et al. (2020)
DIABLO	FC	Any	2019	Singh et al. (2019)
scAI	MD	RNA-seq, epigenomic	2020	Jin et al. (2020)
Seurat v4	NN	Any	2021	Hao et al. (2021)
scMM	DAE	Any	2021	Minoura et al. (2021)
SMILE	DAE	Any	2021	Xu et al. (2022b)
MIRA	TM	RNA-seq, chromatin state	2022	Lynch et al. (2022)

**FIGURE 3:**
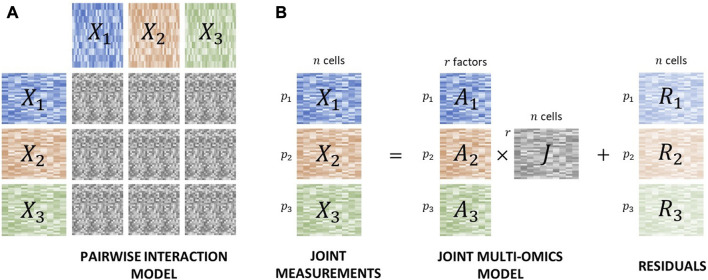
Two main strategies are used for vertical integration of joint assays. **(A)** Local strategies link features across modalities via pairwise correspondence. **(B)** Global strategies link features across modalities via common biological factors.

A family of global VI tools are based on a methodology inspired by canonical correlation analysis (CCA) (Hotelling, 1992), which use joint feature measurements across datasets to identify correlated features across modalities (Figure 3A). RGCCA (Tenenhaus and Tenenhaus, 2011) extended this framework to simultaneously allow the analysis of more than 2 datasets. These concepts have been refined in (Tenenhaus et al., 2014) and DIABLO (Singh et al., 2019) to achieve better feature selection.

On the other hand, other popular global VI tools are based on matrix decomposition algorithms (Figure 3B) (Lock et al., 2013; Argelaguet et al., 2018; 2020; Jin et al., 2020). These tools generally aim to decompose each data matrix into a component explained by global factors, a component containing dataset-specific and modality-specific factors, and a noise term. They mostly differ by their exact decomposition model and specific strategies used to infer its parameters.

If deep autoencoders did wonders for HI, they were also successfully applied to VI problems (Minoura et al., 2021) by using two distinct encoders and decoders using a shared latent space into which both modalities are projected. This strategy notably allows the network to “translate” a modality into another. We can also mention the recent MIRA method (Lynch et al., 2022), which leverages a variational autoencoder approach to learn gene expression and chromatin accessibility shared topics.

Overall, the VI framework has allowed the growth of methods taking advantage of the powerful sample anchoring across datasets, with many approaches proposed inspired by statistics and machine learning. A few important benchmarks have been carried out to assess the quality of VI tools, notably (Cantini et al., 2021) which focuses on joint dimensionality reduction (jDR) methods. Due to the difficulty of setting up joint assays and the inability of these methods to function without matched cells, there is a crucial need for diagonal integration (DI) tools that aim to integrate datasets across batches and modalities.

## 5 Diagonal and mosaic integration jointly embed non- or partially-anchored datasets

**FIGURE 4:**
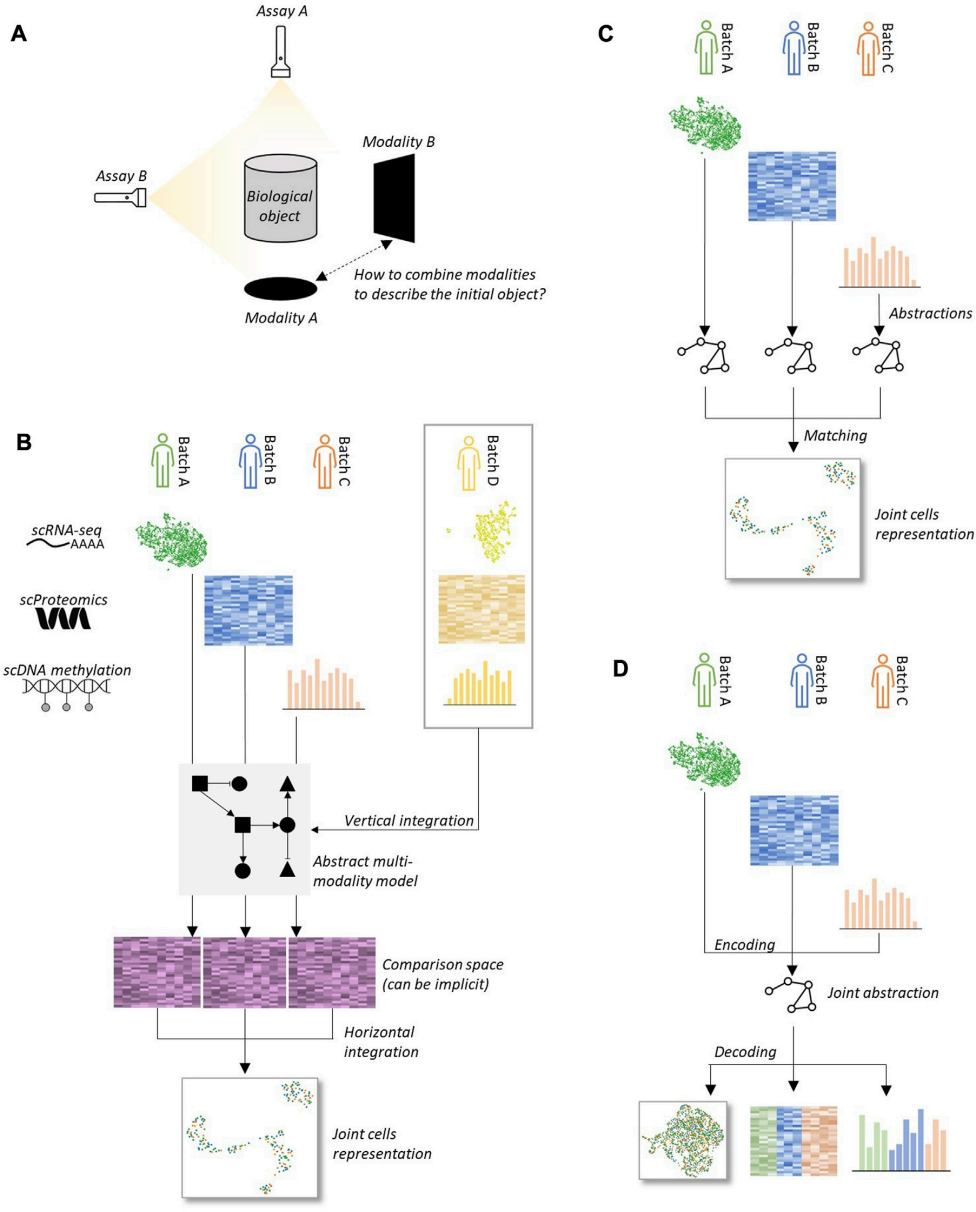
Several strategies can be carried out to tackle the diagonal integration computational challenge **(A)** A biological object (e.g., a population of cells). can be profiled using different assays, without obvious means to link both representations. **(B)** Knowledge of interaction between features across modalities can be obtained from vertical integration of external datasets generated using joint assays. This information can then be leveraged to compare cells between batches even if they are not expressed in the same modality, which allows to use horizontal integration tools. **(C)** Datasets can be independently encoded into abstractions that can then be matched in an unsupervised fashion to build a joint representation of datasets. **(D)** Datasets can be jointly encoded into a unique abstraction, for instance through a learning process using a deep autoencoder framework, that can then be used as a joint embedding of datasets.

Diagonal integration (DI) and mosaic integration (MI) are two data integration frameworks for single-cell data that do not require datasets to be acquired through matched biological assays (Table 3). In this paragraph, we use DI indistinguishably from MI. The goal is to leverage datasets structure and possibly external information, such as genomic locations, pathways, or partial sample or modality overlap to infer complete bonds between cells across modalities without relying on explicit sample anchoring (Figures 4A, B). DI generally aims to build a joint embedding of datasets into a common latent space, while MI focuses on inferring missing modalities from partially anchored datasets. Let us focus on the two main families of methods that exist for tackling DI: manifold alignment and deep autoencoders. These two machine learning paradigms can handle high levels of abstraction, which seems required to tackle DI in the general case.

**TABLE 3:** A non-exhaustive list of diagonal (DI) and mosaic integration (MI) tools that integrate single-cell datasets gathered across different biological samples and modalities. MA, Manifold Alignment; MF, Matrix Factorization; MMD, Maximum Mean Discrepancy; NN, Nearest Neighbors; DAE, Deep Autoencoders; OT, Optimal Transport; GW, Gromov-Wasserstein; LI, Linear Inference.

Tool	Strategy	Input	Output	Year	References
MATCHER	MA	RNA-seq, epigenetic	Gen. Model	2017	Welch et al. (2017)
CoupledNMF	MF	RNA-seq, ATAC-seq	Clustering	2018	Duren et al. (2018)
MMD-MA	MMD	Any	Embedding	2019	Liu et al. (2019)
LIGER	MF	RNA-seq, ATAC-seq, scMethyl	Embedding	2019	Welch et al. (2019)
UnionCom	MA	Any	Embedding	2020	Cao et al. (2020)
bindSC	NN	Any	Embedding	2020	Dou et al. (2020)
SCIM	DAE	Any	Embedding	2020	Stark et al. (2020)
MultiVI	DAE	RNA-seq, ATAC-seq	Embedding	2021	Ashuach et al. (2021)
COBOLT	DAE	Any	Embedding	2021	Gong et al. (2021)
Pamona	OT	Any	Embedding	2022	Cao et al. (2022b)
Polarbear	DAE	RNA-seq, ATAC-seq	Embedding	2022	Zhang et al. (2022a)
GLUE	DAE	Any	Embedding	2022	Cao and Gao (2022)
SCOT	GW	Any	Embedding	2022	Demetci et al. (2022)
scJoint	DAE	RNA-seq, ATAC-seq	Embedding	2022	Lin et al. (2022)
sciCAN	DAE	RNA-seq, ATAC-seq	Embedding	2022	Xu et al. (2022a)
scDART	DAE	RNA-seq, ATAC-seq	Embedding	2022	Zhang et al. (2022b)
StabMap	LI	Any	Embedding	2022	Ghazanfar et al. (2022)
UINMF	MF	RNA-seq, ATAC-seq, spatial	Embedding	2022	Kriebel and Welch (2022)

Manifold alignment methods (Welch et al., 2017; Liu et al., 2019; Cao et al., 2020; Cao et al., 2022b; Demetci et al., 2022) for DI operate similarly as in the HI case and work under the assumption stating that smooth point clouds alignment corresponds to meaningful biological correspondence (Figure 4C). This allows them to work in an unsupervised fashion without requiring additional knowledge other than data matrices. Despite working accurately in some cases, it has been shown this hypothesis is far from being universal (Xu and McCord, 2022). In this article, the authors show that under some simple data tweaking, such as missing cell types or different sample sizes, manifold alignment DI methods can generate erroneous embeddings featuring clusters with mixed cell types. This is concerning, as validating DI is a challenging task, given that it is rarely the case to have reliable cell type labels across modalities at disposal. Therefore, we suggest that these unsupervised manifold alignment methods must be used carefully and only when integration quality control is feasible. In other cases, it is preferable to choose another DI method that allows the user to provide additional information that helps bridge the gap across modalities.

As for HI and VI, deep autoencoders are powerful tools for solving DI tasks, with several advantages. First, they can take advantage of GPU acceleration built in deep learning libraries to greatly speed up the training process, and naturally scale to very large datasets. The second benefit of using these neural networks is that they offer the possibility to train a separate encoder and decoder for each biological modality, which helps capture modality-specific factors compared to manifold alignment algorithms where all omics layers are treated similarly. These separate encoders generally share a joint latent space (Figure 4D), with some form of penalty to force latent representations to overlap. They also present an algorithmic structure that facilitates the introduction of external biological guidance, like in the GLUE tool (Cao and Gao, 2022), which uses a guidance graph as prior knowledge about functional relationships between features across modalities. We would also like to mention in this category the Polarbear tool (Zhang R. et al., 2022), which leverages deep autoencoders to notably translate single-cell data between RNA-seq and ATAC-seq.

To the best of our knowledge, there do not exist at the time of writing a large-scale, independent benchmark of DI methods like for HI (Luecken et al., 2022). This is arguably difficult to set up due to the number of single-cell modalities available today, given the fact that, in addition, not all methods can deal with all modalities. Some may also require specific prior knowledge, and output type may vary. Furthermore, there is a lack of reliable metrics for assessing the quality of DI methods and real-life benchmarking datasets. A first breakthrough is to note in this direction, with a NIPS single-cell analysis competition organized recently which gave access to a public multimodal dataset containing single-cell gene expression, protein expression, and chromatin accessibility using CITE-seq and Multiome (Lance et al., 2022). With the democratization of such datasets, benchmarking DI methods will become more accessible, which will help standardize the field and identify the best-performing methods for each scenario.

To finish, there is a growing interest in integrating single-cell data with other related data modalities, such as whole slide images or spatial transcriptomics. There is a particular interest in deconvoluting spatial transcriptomic spots by integrating them with a single-cell RNA-seq dataset obtained from a similar same tissue. This is a current challenge, and several methods have been proposed for this task, notably benchmarked in (Li et al., 2022).

Overall, DI is arguably the most challenging data integration problem, and solving it is still a very active research area. This very convenient data integration paradigm is extremely versatile, as it theoretically does not need any anchoring (cells or features) between the different datasets. In practice, if many DI tools indeed work in a completely unsupervised way leveraging data topology such as MMD-MA (Liu et al., 2019), Pamona (Cao and Gao, 2022) or SCOT (Demetci et al., 2022), others require additional information to bridge the gap between modalities like GLUE (Cao et al., 2022a) or MultiVI (Ashuach et al., 2021) which can take a covariate design matrix as an optional parameter. For the moment, it appears that these biased methods offer more control on the results, as data topology can be misleading in practice and yield aberrant results (Xu and McCord, 2022). Therefore, using DI tools that can be enriched with biological context seems to be the best choice in the applications where such context can be obtained in a reliable way, typically when integrating datasets where strong covariates exist between modalities.

## 6 Discussion

Data integration consists of distinct challenges depending on the anchoring that exists between datasets, and each facet of DI requires distinct tools that leverage various algorithmic strategies. For instance, metric-based methods excel at solving HI tasks, whereas linear matrix analysis methods excel at solving VI tasks. Machine learning paradigms with high abstraction levels, such as manifold alignment methods and deep neural networks, are excellent assets for dealing with DI and MI problems, the latter also performing well at HI and VI tasks. Overall, VI methods are pretty good at solving the task, HI methods are capable of dealing with small to moderate batch effects but still struggle to mitigate significant batch effects such as inter-species data, and DI/MI problems are arguably still unsolved in the general case.

We talked about the Transmorph framework that articulates computational blocks to conceive HI pipelines, but this is not the only framework that exists which is related to data integration. We can cite MUON (Bredikhin et al., 2022), which facilitates the handling of data consisting of different modalities, Polyphony (Cheng et al., 2022), which carries out transfer learning across datasets by leveraging data integration algorithms, or SinCast (Deng et al., 2022) which is specialized in cell type inference by mapping a query onto an atlas.

It is essential to note that there are important pitfalls to data integration that must not be overlooked. The primary issue that can be encountered is named *overcorrection* and describes an undesirable event where a data integration method incorrectly aligns cells that do not share the same biological type or state. This typically happens when batch effects are too strong, when a dataset contains specific cell types, when cell type distribution is highly imbalanced, or when there is little anchoring between batches. Overcorrection can be difficult to detect when there is no easy access to cell labels and is a critical issue that hinder every subsequent analysis step. Indeed, it can lead to cells belonging to the same cluster without sharing critical biological properties such as cell type or states. Other issues are worth noting even though they are not exclusive to the data integration task, such as the difficulty in differentiating between true zeros and missing values in RNA-seq datasets or the fact that different modalities are often expressed using different data types (e.g., binary or integer data) which may be difficult to handle jointly within mathematical frameworks. Finally, data integration tools based on abstract machine learning paradigms such as deep autoencoders often comes at the cost of a decrease in model interpretability which is an important downside for any health-related application. However, many efforts are made to overcome this issue (Svensson et al., 2020; Treppner et al., 2022) and we expect to see many more in the years to come.

There is always an urgent need for large-scale, independent benchmarks like the HI benchmark proposed in (Luecken et al., 2022), or the VI benchmark carried out in (Cantini et al., 2021). To the best of our knowledge, there is still a lack of large-scale independent DI and MI benchmarks. Two things are necessary to carry out such benchmarks: high-quality datasets and reliable metrics. A list of potential datasets can be found in (Argelaguet et al., 2021). There is no clear consensus about which quality assessment metric to use, and most benchmarks like (Luecken et al., 2022) opt for a mixture of metrics that cover several aspects of data integration: conservation of biological variance (CBV) metrics which measure how close similar cells (type or state) are after integration, and removal of batch effects (RBE) metrics. Some CBV metrics are label-based, such as normalized mutual information (NMI), adjusted Rand index (ARI), average silhouette width (ASW), class local inverse Simpson’s index (cLISI), isolated label F1 (ILF) and isolated label silhouette (ILS), others are label-free and generally assess the conservation of biological processes such as cell cycle, highly variable genes, and transcriptomic trajectories. RBE metrics include batch-PC regression, batch-ASW, graph connectivity, iLISI, and kBet. We often observe a tradeoff between CBV and RBE, which can lead to different methods choice depending on the application, whether it is preferable to have good dataset mixing or conservation of subtle biological signals.

To conclude, years of algorithmic and computational advances made it possible to solve most HI and VI problems with satisfying performance, with only the most complicated instances still being problematic (e.g., HI of many batches with strong batch effects). Solving DI and MI is the next computational challenge. The most promising approaches that have been developed to tackle it are based on deep learning models, particularly deep autoencoders. It has been shown that purely unsupervised DI may not be a well-posed problem and could suffer fundamental flaws (Xu and McCord, 2022), which greatly incentivizes using knowledge-driven tools that allow the user to include external information to enhance models with functional information that link features across modalities. Finally, apart from developing new tools, there is also an urgent need to enrich the data integration ecosystem with organizing frameworks, standardized benchmarks, datasets, and quality assessment metrics.

## References

[B1] Abdelaal T. Mourragui S. Mahfouz A. Reinders M. J. (2020). Spage: Spatial gene enhancement using scrna-seq. Nucleic acids Res. 48, e107. 10.1093/nar/gkaa740 32955565 PMC7544237

[B2] Aebersold R. Mann M. (2003). Mass spectrometry-based proteomics. Nature 422, 198–207. 10.1038/nature01511 12634793

[B3] Anaissi A. Zandavi S. M. Suleiman B. Alyassine W. Braytee A. Vafaee F. (2022). A benchmark of pre-processing effect on single cell RNA sequencing integration methods. Preprint, In Review. 10.21203/rs.3.rs-2249309/v1

[B4] Angermueller C. Clark S. J. Lee H. J. Macaulay I. C. Teng M. J. Hu T. X. (2016). Parallel single-cell sequencing links transcriptional and epigenetic heterogeneity. Nat. methods 13, 229–232. 10.1038/nmeth.3728 26752769 PMC4770512

[B5] Argelaguet R. Arnol D. Bredikhin D. Deloro Y. Velten B. Marioni J. C. (2020). MOFA+: A statistical framework for comprehensive integration of multi-modal single-cell data. Genome Biol. 21, 111. 10.1186/s13059-020-02015-1 32393329 PMC7212577

[B6] Argelaguet R. Cuomo A. S. Stegle O. Marioni J. C. (2021). Computational principles and challenges in single-cell data integration. Nat. Biotechnol. 39, 1202–1215. 10.1038/s41587-021-00895-7 33941931

[B7] Argelaguet R. Velten B. Arnol D. Dietrich S. Zenz T. Marioni J. C. (2018). Multi-omics factor analysis—A framework for unsupervised integration of multi-omics data sets. Mol. Syst. Biol. 14, e8124. 10.15252/msb.20178124 29925568 PMC6010767

[B8] Ashuach T. Gabitto M. I. Jordan M. I. Yosef N. (2021). MultiVI: Deep generative model for the integration of multi-modal data. Bioinformatics 2021. 10.1101/2021.08.20.457057 PMC10406609 37386189

[B9] Barkas N. Petukhov V. Nikolaeva D. Lozinsky Y. Demharter S. Khodosevich K. (2019). Joint analysis of heterogeneous single-cell rna-seq dataset collections. Nat. methods 16, 695–698. 10.1038/s41592-019-0466-z 31308548 PMC6684315

[B10] Biancalani T. Scalia G. Buffoni L. Avasthi R. Lu Z. Sanger A. (2021). Deep learning and alignment of spatially resolved single-cell transcriptomes with tangram. Nat. methods 18, 1352–1362. 10.1038/s41592-021-01264-7 34711971 PMC8566243

[B11] Bredikhin D. Kats I. Stegle O. (2022). MUON: Multimodal omics analysis framework. Genome Biol. 23, 42. 10.1186/s13059-021-02577-8 35105358 PMC8805324

[B12] Buenrostro J. D. Wu B. Chang H. Y. Greenleaf W. J. (2015a). Atac-seq: A method for assaying chromatin accessibility genome-wide. Curr. Protoc. Mol. Biol. 109, 21. 10.1002/0471142727.mb2129s109 PMC4374986 25559105

[B13] Buenrostro J. D. Wu B. Litzenburger U. M. Ruff D. Gonzales M. L. Snyder M. P. (2015b). Single-cell chromatin accessibility reveals principles of regulatory variation. Nature 523, 486–490. 10.1038/nature14590 26083756 PMC4685948

[B14] Butler A. Hoffman P. Smibert P. Papalexi E. Satija R. (2018). Integrating single-cell transcriptomic data across different conditions, technologies, and species. Nat. Biotechnol. 36, 411–420. 10.1038/nbt.4096 29608179 PMC6700744

[B15] Camps J. Noël F. Liechti R. Massenet-Regad L. Rigade S. Götz L. (2023). Meta-analysis of human cancer single-cell rna-seq datasets using the immucan database. Cancer Res. 83, 363–373. 10.1158/0008-5472.can-22-0074 36459564 PMC9896021

[B16] Cantini L. Zakeri P. Hernandez C. Naldi A. Thieffry D. Remy E. (2021). Benchmarking joint multi-omics dimensionality reduction approaches for the study of cancer. Nat. Commun. 12, 124. 10.1038/s41467-020-20430-7 33402734 PMC7785750

[B17] Cao K. Bai X. Hong Y. Wan L. (2020). Unsupervised topological alignment for single-cell multi-omics integration. Bioinformatics 36, i48–i56. 10.1093/bioinformatics/btaa443 32657382 PMC7355262

[B18] Cao K. Gong Q. Hong Y. Wan L. (2022a). A unified computational framework for single-cell data integration with optimal transport. Nat. Commun. 13, 7419. 10.1038/s41467-022-35094-8 36456571 PMC9715710

[B19] Cao K. Hong Y. Wan L. (2022b). Manifold alignment for heterogeneous single-cell multi-omics data integration using pamona. Bioinformatics 38, 211–219. 10.1093/bioinformatics/btab594 PMC8696097 34398192

[B20] Cao Z.-J. Gao G. (2022). Multi-omics single-cell data integration and regulatory inference with graph-linked embedding. Nat. Biotechnol. 40, 1458–1466. 10.1038/s41587-022-01284-4 35501393 PMC9546775

[B21] Castanedo F. (2013). A review of data fusion techniques. Sci. world J. 2013, 1–19. 10.1155/2013/704504 PMC3826336 24288502

[B22] Chen H. Albergante L. Hsu J. Y. Lareau C. A. Lo Bosco G. Guan J. (2019a). Single-cell trajectories reconstruction, exploration and mapping of omics data with stream. Nat. Commun. 10, 1903. 10.1038/s41467-019-09670-4 31015418 PMC6478907

[B23] Chen S. Lake B. B. Zhang K. (2019b). High-throughput sequencing of the transcriptome and chromatin accessibility in the same cell. Nat. Biotechnol. 37, 1452–1457. 10.1038/s41587-019-0290-0 31611697 PMC6893138

[B24] Cheng F. Keller M. S. Qu H. Gehlenborg N. Wang Q. (2022). Polyphony: An interactive transfer learning framework for single-cell data analysis. IEEE Trans. Vis. Comput. Graph 29, 591. 10.1109/TVCG.2022.3209408 36155452 PMC10039961

[B25] Cheow L. F. Courtois E. T. Tan Y. Viswanathan R. Xing Q. Tan R. Z. (2016). Single-cell multimodal profiling reveals cellular epigenetic heterogeneity. Nat. methods 13, 833–836. 10.1038/nmeth.3961 27525975

[B26] Conesa A. Beck S. (2019). Making multi-omics data accessible to researchers. Sci. data 6, 251. 10.1038/s41597-019-0258-4 31672978 PMC6823467

[B27] Cuomo A. S. Seaton D. D. McCarthy D. J. Martinez I. Bonder M. J. Garcia-Bernardo J. (2020). Single-cell rna-sequencing of differentiating ips cells reveals dynamic genetic effects on gene expression. Nat. Commun. 11, 810. 10.1038/s41467-020-14457-z 32041960 PMC7010688

[B28] Demetci P. Santorella R. Sandstede B. Noble W. S. Singh R. (2022). Scot: Single-cell multi-omics alignment with optimal transport. J. Comput. Biol. 29, 3–18. 10.1089/cmb.2021.0446 35050714 PMC8812493

[B29] Deng Y. Choi J. Lê Cao K.-A. (2022). Sincast: A computational framework to predict cell identities in single-cell transcriptomes using bulk atlases as references. Briefings Bioinforma. 23, bbac088. 10.1093/bib/bbac088 PMC9155616 35362513

[B30] Dong W. Moses C. Li K. (2011). “Efficient k-nearest neighbor graph construction for generic similarity measures,” in Proceedings of the 20th international conference on World wide web, Hyderabad, India, March 28 - April 01, 2011, 577–586.

[B31] Dou J. Liang S. Mohanty V. Cheng X. Kim S. Choi J. (2020). Unbiased integration of single cell multi-omics data. BioRxiv.

[B32] Duren Z. Chen X. Zamanighomi M. Zeng W. Satpathy A. T. Chang H. Y. (2018). Integrative analysis of single-cell genomics data by coupled nonnegative matrix factorizations. Proc. Natl. Acad. Sci. 115, 7723–7728. 10.1073/pnas.1805681115 29987051 PMC6065048

[B33] Eraslan G. Simon L. M. Mircea M. Mueller N. S. Theis F. J. (2019). Single-cell rna-seq denoising using a deep count autoencoder. Nat. Commun. 10, 390–414. 10.1038/s41467-018-07931-2 30674886 PMC6344535

[B34] Eto M. Hirota W. Seno S. Matsuda H. (2018). “Asymmetric integration of single-cell transcriptomic data using latent dirichlet allocation and procrustes analysis,” in 2018 IEEE International Conference on Bioinformatics and Biomedicine (BIBM) (IEEE), Madrid, Spain, Dec. 3 2018 to Dec. 6 2018, 2129. –2135.

[B35] Farahani A. Voghoei S. Rasheed K. Arabnia H. R. (2021). A brief review of domain adaptation. Adv. data Sci. Inf. Eng. 2021, 877–894. 10.1007/978-3-030-71704-9_65

[B36] Fouché A. Chadoutaud L. Delattre O. Zinovyev A. (2022). transmorph: a unifying computational framework for single-cell data integration. bioRxiv 10.1093/nargab/lqad069 PMC10336778 37448589

[B37] Gao J. Li P. Chen Z. Zhang J. (2020). A survey on deep learning for multimodal data fusion. Neural Comput. 32, 829–864. 10.1162/neco_a_01273 32186998

[B38] Ghazanfar S. Guibentif C. Marioni J. C. (2022). Stabmap: Mosaic single cell data integration using non-overlapping features. bioRxiv, 2022–2102. 10.1038/s41587-023-01766-z PMC10869270 37231260

[B39] Gong B. Zhou Y. Purdom E. (2021). Cobolt: Integrative analysis of multimodal single-cell sequencing data. Genome Biol. 22, 351–421. 10.1186/s13059-021-02556-z 34963480 PMC8715620

[B40] Gower J. C. (1975). Generalized procrustes analysis. Psychometrika 40, 33–51. 10.1007/bf02291478

[B41] Guo H. Zhu P. Wu X. Li X. Wen L. Tang F. (2013). Single-cell methylome landscapes of mouse embryonic stem cells and early embryos analyzed using reduced representation bisulfite sequencing. Genome Res. 23, 2126–2135. 10.1101/gr.161679.113 24179143 PMC3847781

[B42] Haghverdi L. Lun A. T. L. Morgan M. D. Marioni J. C. (2018). Batch effects in single-cell RNA-sequencing data are corrected by matching mutual nearest neighbors. Nat. Biotechnol. 36, 421–427. 10.1038/nbt.4091 29608177 PMC6152897

[B43] Hao Y. Hao S. Andersen-Nissen E. Mauck W. M. III Zheng S. Butler A. (2021). Integrated analysis of multimodal single-cell data. Cell 184, 3573–3587.e29. 10.1016/j.cell.2021.04.048 34062119 PMC8238499

[B44] Hie B. Bryson B. Berger B. (2019). Efficient integration of heterogeneous single-cell transcriptomes using scanorama. Nat. Biotechnol. 37, 685–691. 10.1038/s41587-019-0113-3 31061482 PMC6551256

[B45] Hotelling H. (1992). “Relations between two sets of variates,” in Breakthroughs in statistics: Methodology and distribution. Editors Kotz S. Johnson N. L. (New York, NY: Springer). 10.1007/978-1-4612-4380-9_14

[B46] Jin S. Zhang L. Nie Q. (2020). scAI: an unsupervised approach for the integrative analysis of parallel single-cell transcriptomic and epigenomic profiles. Genome Biol. 21, 25. 10.1186/s13059-020-1932-8 32014031 PMC6996200

[B47] Johansen N. Quon G. (2019). scAlign: a tool for alignment, integration, and rare cell identification from scRNA-seq data. Genome Biol. 20, 166. 10.1186/s13059-019-1766-4 31412909 PMC6693154

[B48] Johnson W. E. Li C. Rabinovic A. (2007). Adjusting batch effects in microarray expression data using empirical bayes methods. Biostatistics 8, 118–127. 10.1093/biostatistics/kxj037 16632515

[B49] Tabula Sapiens Consortium, Jones R. C. Karkanias J. Krasnow M. A. Pisco A. O. Quake S. R. (2022). The tabula sapiens: A multiple-organ, single-cell transcriptomic atlas of humans. Science 376, eabl4896. 10.1126/science.abl4896 35549404 PMC9812260

[B50] Kiselev V. Y. Yiu A. Hemberg M. (2018). scmap: projection of single-cell RNA-seq data across data sets. Nat. Methods 15, 359–362. 10.1038/nmeth.4644 29608555

[B51] Klein A. M. Mazutis L. Akartuna I. Tallapragada N. Veres A. Li V. (2015). Droplet barcoding for single-cell transcriptomics applied to embryonic stem cells. Cell 161, 1187–1201. 10.1016/j.cell.2015.04.044 26000487 PMC4441768

[B52] Korsunsky I. Millard N. Fan J. Slowikowski K. Zhang F. Wei K. (2019). Fast, sensitive and accurate integration of single-cell data with Harmony. Nat. Methods 16, 1289–1296. 10.1038/s41592-019-0619-0 31740819 PMC6884693

[B53] Kriebel A. R. Welch J. D. (2022). Uinmf performs mosaic integration of single-cell multi-omic datasets using nonnegative matrix factorization. Nat. Commun. 13, 780–817. 10.1038/s41467-022-28431-4 35140223 PMC8828882

[B54] La Manno G. Soldatov R. Zeisel A. Braun E. Hochgerner H. Petukhov V. (2018). Rna velocity of single cells. Nature 560, 494–498. 10.1038/s41586-018-0414-6 30089906 PMC6130801

[B55] Lance C. Luecken M. D. Burkhardt D. B. Cannoodt R. Rautenstrauch P. Laddach A. C. (2022). Multimodal single cell data integration challenge: Results and lessons learned. bioRxiv.

[B56] Li B. Zhang W. Guo C. Xu H. Li L. Fang M. (2022). Benchmarking spatial and single-cell transcriptomics integration methods for transcript distribution prediction and cell type deconvolution. Nat. Methods 19, 662–670. 10.1038/s41592-022-01480-9 35577954

[B57] Li X. Wang K. Lyu Y. Pan H. Zhang J. Stambolian D. (2020). Deep learning enables accurate clustering with batch effect removal in single-cell rna-seq analysis. Nat. Commun. 11, 2338–2414. 10.1038/s41467-020-15851-3 32393754 PMC7214470

[B58] Lin Y. Wu T.-Y. Wan S. Yang J. Y. Wong W. H. Wang Y. R. (2022). Scjoint integrates atlas-scale single-cell rna-seq and atac-seq data with transfer learning. Nat. Biotechnol. 40, 703–710. 10.1038/s41587-021-01161-6 35058621 PMC9186323

[B59] Liu J. Huang Y. Singh R. Vert J.-P. Noble W. S. (2019). Jointly embedding multiple single-cell omics measurements. Algorithms Bioinform 143, 10. 10.4230/LIPIcs.WABI.2019.10 34632462 PMC8496402

[B60] Lock E. F. Hoadley K. A. Marron J. Nobel A. B. (2013). Joint and individual variation explained (jive) for integrated analysis of multiple data types. Ann. Appl. Stat. 7, 523–542. 10.1214/12-AOAS597 23745156 PMC3671601

[B61] Lopez R. Regier J. Cole M. B. Jordan M. I. Yosef N. (2018). Deep generative modeling for single-cell transcriptomics. Nat. Methods 15, 1053–1058. 10.1038/s41592-018-0229-2 30504886 PMC6289068

[B62] Loza M. Teraguchi S. Standley D. M. Diez D. (2022). Unbiased integration of single cell transcriptome replicates. NAR Genomics Bioinforma. 4, lqac022. lqac022. 10.1093/nargab/lqac022 PMC8923008 35300461

[B63] Luecken M. D. Büttner M. Chaichoompu K. Danese A. Interlandi M. Mueller M. F. (2022). Benchmarking atlas-level data integration in single-cell genomics. Nat. Methods 19, 41–50. 10.1038/s41592-021-01336-8 34949812 PMC8748196

[B64] Lynch A. W. Theodoris C. V. Long H. W. Brown M. Liu X. S. Meyer C. A. (2022). MIRA: Joint regulatory modeling of multimodal expression and chromatin accessibility in single cells. Nat. Methods 19, 1097–1108. 10.1038/s41592-022-01595-z 36068320 PMC9517733

[B65] Macosko E. Z. Basu A. Satija R. Nemesh J. Shekhar K. Goldman M. (2015). Highly parallel genome-wide expression profiling of individual cells using nanoliter droplets. Cell 161, 1202–1214. 10.1016/j.cell.2015.05.002 26000488 PMC4481139

[B66] Minoura K. Abe K. Nam H. Nishikawa H. Shimamura T. (2021). A mixture-of-experts deep generative model for integrated analysis of single-cell multiomics data. Cell Rep. methods 1, 100071. 10.1016/j.crmeth.2021.100071 35474667 PMC9017195

[B67] Mirkes E. M. Bac J. Fouché A. Stasenko S. V. Zinovyev A. Gorban A. N. (2023). Domain adaptation principal component analysis: Base linear method for learning with out-of-distribution data. Entropy 25, 33. 10.3390/e25010033 PMC9858254 36673174

[B68] Pan S. J. Tsang I. W. Kwok J. T. Yang Q. (2010). Domain adaptation via transfer component analysis. IEEE Trans. neural Netw. 22, 199–210. 10.1109/tnn.2010.2091281 21095864

[B69] Pantanowitz L. Valenstein P. N. Evans A. J. Kaplan K. J. Pfeifer J. D. Wilbur D. C. (2011). Review of the current state of whole slide imaging in pathology. J. pathology Inf. 2, 36. 10.4103/2153-3539.83746 PMC3162745 21886892

[B70] Polański K. Young M. D. Miao Z. Meyer K. B. Teichmann S. A. Park J.-E. (2020). BBKNN: Fast batch alignment of single cell transcriptomes. Bioinformatics 36, 964–965. 10.1093/bioinformatics/btz625 31400197 PMC9883685

[B71] Satija R. Farrell J. A. Gennert D. Schier A. F. Regev A. (2015). Spatial reconstruction of single-cell gene expression data. Nat. Biotechnol. 33, 495–502. 10.1038/nbt.3192 25867923 PMC4430369

[B72] Schaum N. Karkanias J. Neff N. F. May A. P. Quake S. R. Wyss-Coray T. (2018). Single-cell transcriptomics of 20 mouse organs creates a tabula muris: The tabula muris consortium. Nature 562, 367–372. 10.1038/s41586-018-0590-4 30283141 PMC6642641

[B73] Schiebinger G. Shu J. Tabaka M. Cleary B. Subramanian V. Solomon A. (2019). Optimal-transport analysis of single-cell gene expression identifies developmental trajectories in reprogramming. Cell 176, 928–943.e22. 10.1016/j.cell.2019.01.006 30712874 PMC6402800

[B74] Singh A. Shannon C. P. Gautier B. Rohart F. Vacher M. Tebbutt S. J. (2019). Diablo: An integrative approach for identifying key molecular drivers from multi-omics assays. Bioinformatics 35, 3055–3062. 10.1093/bioinformatics/bty1054 30657866 PMC6735831

[B75] Ståhl P. L. Salmén F. Vickovic S. Lundmark A. Navarro J. F. Magnusson J. (2016). Visualization and analysis of gene expression in tissue sections by spatial transcriptomics. Science 353, 78–82. 10.1126/science.aaf2403 27365449

[B76] Stark S. G. Ficek J. Locatello F. Bonilla X. Chevrier S. Singer F. (2020). Scim: Universal single-cell matching with unpaired feature sets. Bioinformatics 36, i919–i927. 10.1093/bioinformatics/btaa843 33381818 PMC7773480

[B77] Stoeckius M. Hafemeister C. Stephenson W. Houck-Loomis B. Chattopadhyay P. K. Swerdlow H. (2017). Simultaneous epitope and transcriptome measurement in single cells. Nat. methods 14, 865–868. 10.1038/nmeth.4380 28759029 PMC5669064

[B78] Stuart T. Butler A. Hoffman P. Hafemeister C. Papalexi E. Mauck W. M. (2019). Comprehensive integration of single-cell data. Cell 177, 1888–1902.e21. 10.1016/j.cell.2019.05.031 31178118 PMC6687398

[B79] Sugihara R. Kato Y. Mori T. Kawahara Y. (2022). Alignment of single-cell trajectory trees with CAPITAL. Nat. Commun. 13, 5972. 10.1038/s41467-022-33681-3 36241645 PMC9568509

[B80] Sun D. Guan X. Moran A. E. Wu L.-Y. Qian D. Z. Schedin P. (2022). Identifying phenotype-associated subpopulations by integrating bulk and single-cell sequencing data. Nat. Biotechnol. 40, 527–538. 10.1038/s41587-021-01091-3 34764492 PMC9010342

[B81] Svensson V. Gayoso A. Yosef N. Pachter L. (2020). Interpretable factor models of single-cell rna-seq via variational autoencoders. Bioinformatics 36, 3418–3421. 10.1093/bioinformatics/btaa169 32176273 PMC7267837

[B82] Tenenhaus A. Philippe C. Guillemot V. Le Cao K.-A. Grill J. Frouin V. (2014). Variable selection for generalized canonical correlation analysis. Biostatistics 15, 569–583. 10.1093/biostatistics/kxu001 24550197

[B83] Tenenhaus A. Tenenhaus M. (2011). Regularized generalized canonical correlation analysis. Psychometrika 76, 257–284. 10.1007/s11336-011-9206-8 28536930

[B84] Tibes R. Qiu Y. Lu Y. Hennessy B. Andreeff M. Mills G. B. (2006). Reverse phase protein array: Validation of a novel proteomic technology and utility for analysis of primary leukemia specimens and hematopoietic stem cells. Mol. cancer Ther. 5, 2512–2521. 10.1158/1535-7163.mct-06-0334 17041095

[B85] Tran H. T. N. Ang K. S. Chevrier M. Zhang X. Lee N. Y. S. Goh M. (2020). A benchmark of batch-effect correction methods for single-cell rna sequencing data. Genome Biol. 21, 12–32. 10.1186/s13059-019-1850-9 31948481 PMC6964114

[B86] Treppner M. Binder H. Hess M. (2022). Interpretable generative deep learning: An illustration with single cell gene expression data. Hum. Genet. 141, 1481–1498. 10.1007/s00439-021-02417-6 34988661 PMC9360114

[B87] Trong T. N. Mehtonen J. González G. Kramer R. Hautamäki V. Heinäniemi M. (2020). Semisupervised generative autoencoder for single-cell data. J. Comput. Biol. 27, 1190–1203. 10.1089/cmb.2019.0337 31794242 PMC7415880

[B88] Van Der Wijst M. G. Brugge H. De Vries D. H. Deelen P. Swertz M. A. Study L. C. (2018). Single-cell rna sequencing identifies celltype-specific cis-eqtls and co-expression qtls. Nat. Genet. 50, 493–497. 10.1038/s41588-018-0089-9 29610479 PMC5905669

[B89] Wang C. Krafft P. Mahadevan S. Ma Y. Fu Y. (2011). “Manifold alignment,” in Manifold Learning: Theory and Applications, 95–120.

[B90] Wang D. Gu J. (2018). Vasc: Dimension reduction and visualization of single-cell rna-seq data by deep variational autoencoder. Genomics, proteomics Bioinforma. 16, 320–331. 10.1016/j.gpb.2018.08.003 PMC6364131 30576740

[B91] Weinstein J. Collisson E. Mills G. Mills Shaw K. Ozenberger B. Ellrott K. (2013). The cancer genome atlas pan-cancer analysis project. Nat. Genet. 45, 1113–1120. 10.1038/ng.2764 24071849 PMC3919969

[B92] Welch J. D. Hartemink A. J. Prins J. F. (2017). MATCHER: Manifold alignment reveals correspondence between single cell transcriptome and epigenome dynamics. Genome Biol. 18, 138. 10.1186/s13059-017-1269-0 28738873 PMC5525279

[B93] Welch J. D. Kozareva V. Ferreira A. Vanderburg C. Martin C. Macosko E. Z. (2019). Single-cell multi-omic integration compares and contrasts features of brain cell identity. Cell 177, 1873–1887.e17. 10.1016/j.cell.2019.05.006 31178122 PMC6716797

[B94] Westermeier R. Marouga R. (2005). Protein detection methods in proteomics research. Biosci. Rep. 25, 19–32. 10.1007/s10540-005-2845-1 16222417

[B95] Wolf F. A. Angerer P. Theis F. J. (2018). Scanpy: Large-scale single-cell gene expression data analysis. Genome Biol. 19, 15–5. 10.1186/s13059-017-1382-0 29409532 PMC5802054

[B96] Xu Y. Begoli E. McCord R. P. (2022a). sciCAN: single-cell chromatin accessibility and gene expression data integration via cycle-consistent adversarial network. npj Syst. Biol. Appl. 8, 33–10. 10.1038/s41540-022-00245-6 36089620 PMC9464763

[B97] Xu Y. Das P. McCord R. P. (2022b). SMILE: Mutual information learning for integration of single-cell omics data. Bioinformatics 38, 476–486. 10.1093/bioinformatics/btab706 34623402 PMC10060712

[B98] Xu Y. McCord R. P. (2022). Diagonal integration of multimodal single-cell data: Potential pitfalls and paths forward. Nat. Commun. 13, 3505. 10.1038/s41467-022-31104-x 35717437 PMC9206644

[B99] You K. Long M. Cao Z. Wang J. Jordan M. I. (2019). “Universal domain adaptation,” in Proceedings of the IEEE/CVF conference on computer vision and pattern recognition, Long Beach, CA, USA, June 15 2019 to June 20 2019, 2720.

[B100] Zhang R. Meng-Papaxanthos L. Vert J.-p. Noble W. S. (2022a). Multimodal single-cell translation and alignment with semi-supervised learning. J. Comput. Biol. 29, 1198–1212. 10.1089/cmb.2022.0264 36251758 PMC9700358

[B101] Zhang Z. Yang C. Zhang X. (2022b). scDART: integrating unmatched scRNA-seq and scATAC-seq data and learning cross-modality relationship simultaneously. Genome Biol. 23, 139. 10.1186/s13059-022-02706-x 35761403 PMC9238247

